# Introduction to the Physics of Ionic Conduction in Narrow Biological and Artificial Channels

**DOI:** 10.3390/e23060644

**Published:** 2021-05-21

**Authors:** Dmitry G. Luchinsky, Peter V. E. McClintock

**Affiliations:** 1 Department of Physics, Lancaster University, Lancaster LA1 4YB, UK; d.luchinsky@lancaster.ac.uk; 2 KBR Inc., Ames Research Center, Moffett Field, CA 94035, USA

“There is plenty of room at the bottom”

Richard Feynman

The permeation of ions through narrow water-filled channels is essential to life and of rapidly growing importance in technology. Reaching an understanding of the mechanisms underlying the permeation process requires an interdisciplinary approach, where ideas drawn from physics are of particular importance and have brought encouraging progress in recent years. This introduction sets into context the several ground-breaking papers presented in the *Entropy* Special Issue on “The Physics of Ionic Conduction in Narrow Biological and Artificial Channels”.

Understanding, predicting and optimising the ionic selective transport properties of nanopores remains a critical challenge, both to nanotechnology and to biophysics. The last few decades have witnessed substantial progress in the analysis of such transport based on the use of a variety of experimental, numerical, and theoretical methods. Indeed, it would require several books to do full justice to the current state of the art in the field.

In some cases, the crystal structures (e.g., those of potassium, sodium, and calcium voltage-gated channels) have been discovered. This has provided invaluable insight, but has also thrown into sharp relief the structure–function problem: how to predict the conduction/selectivity properties of a known structure; or, conversely, how to design a structure with the required properties. A reliable solution to the problem promises to open new horizons in terms of pharmaceutical applications and the improved fabrication of solid-state nanopores for the sensing of molecules, desalination, DNA sequencing, and the other developments that together are marking a new era in nanotechnology.

Novel numerical methods and computer hardware now enable microsecond-long simulations of systems with hundreds thousands of atoms and the exploration of polarisable and quantum mechanical force fields. They provide unprecedented capabilities for reaching an understanding of experimental data and for the development of novel devices and techniques. Theoretical advances not only underlie many developments in molecular dynamics, including enhanced sampling and advanced force fields, but are also opening up new research frontiers and shedding fresh light on a number of longstanding problems such as binding probabilities, knock-on mechanisms of conduction, gating, electric double-layers, and local dielectric permittivity, just to mention a few.

It is now appreciated that selective conduction in biological ion channels has a great deal in common with that in artificial nanopores. In each case, there are intriguing analogies with the physics of quantum dots leading to the development of the theory of ionic Coulomb blockade. We dedicate this Issue to the memory of our late colleague, Dr Igor Kh. Kaufman, who developed an elegant theory of ionic Coulomb blockade in biological ion channels and suggested a simple classification of voltage-gated channels based on the charge of the selectivity filter.

At the same time, it is known that specific features of ionic conduction (e.g., dehydration, ion-specific binding affinities, protonation, the multicomponent and competitive nature of ion dynamics, the complex and adaptive structure of the ionic pathway, long-range interaction, local variation of the effective dielectric constant, highly correlated motion of more than one ion within a narrow channel, electric double layers, and water layering at the channel entrances) add many layers of complexity to the fundamental physics analogies.

This Special Issue brings together original high-quality papers on ionic permeation through narrow water-filled channels, both biological and artificial, from some of the best researchers in the field. It includes papers on the statistical physics of the process, on molecular dynamics and Brownian dynamics simulations, and on relevant experiments. Although any selection of papers can only be a narrow slice of the field, our aim is to emphasise the complexity and mutual interdependence of recent multifaceted progress in understanding the physics of ion channels and nanopores. The time is ripe for bringing together these complementary approaches, and we anticipate that they will facilitate major breakthroughs, enabling the design of nanopores to meet particular technological requirements as well as improvements in drug design and perhaps in personalised medicine.

Importantly, the Poisson–Nernst–Planck (PNP) and kinetic models remain among the principal tools for predicting current through nanopores, both in biology and nanotechnology. An example of the classical application of the PNP model to the analysis of reversal potentials and zero-current fluxes, in a system with a fixed profile of permanent charges and two mobile ion species, is provided by the paper by Mofidi et al. [1]. Rigorous analytic and numerical results establish the dependence of the electric and chemical potential profiles on voltage and permanent charge.

At the same time, it is well known that classical Poisson–Boltzmann (PB) and PNP theories do not take account of short-range ion–ion, ion–wall, or ion–water interactions in ion channels. Efforts to eliminate or ameliorate the effects of this deficiency of the continuum models have a long history. This stream of research is represented by the interesting paper of J.-L. Liu and R.S. Eisenberg [2], featuring the development of a molecular mean-field theory—a fourth-order Poisson–Nernst–Planck–Bikerman theory for modelling ionic and water flows in biological ion channels. The theory treats ions and water molecules, in channels of any volume or shape, with interstitial voids, polarisation of water, and ion–ion and ion–water correlations. It can be applied to electrolyte solutions in the nanopores of batteries and fuel cells.

The modelling of ionic currents with reduced models is extensively analysed by Boda et al. [3]. They show that channels are especially amenable to reduced modelling because their functions and the relationships between input parameters (e.g., applied voltage, bath concentrations) and output parameters (e.g., current, rectification, selectivity) are well-defined, allowing one to focus on the physics of input–output relationships rather than on the atomic-scale physics inside the pore. Based on decades of research, the authors propose four general rules for constructing good reduced models of ion channels and nanopores, focusing on the physics of input–output relationships rather than on atomic structure. The proposed rules relate to the importance of (1) the axial concentration profiles, (2) the pore charges, (3) choosing the right explicit degrees of freedom, and (4) creating the proper response functions. Examples demonstrating the application of these rules are provided. Further improvements in predicting the capabilities of reduced models can be achieved by incorporating into the solution of the one-dimensional electro-diffusion model the potential of the mean force obtained from MD simulations. The performance of two such methods is examined by A. Pohorille and M. A. Wilson1 [4] using stochastic simulations. These methods require neither knowledge of the diffusivity nor simulations at multiple voltages, which greatly reduces the computational effort needed to probe the electrophysiology of ion channels. They can be used to determine the free energy profiles from either forward or backward one-sided properties of ions in the channel, such as ion fluxes, density profiles, committor probabilities, or from their two-sided combination. In this work, large sets of stochastic trajectories were generated, individually designed to mimic the molecular dynamics crossing statistics of models for channels of trichotoxin, p7 from hepatitis C and a bacterial homolog of the pentameric ligand-gated ion channel (LGIC). The authors found that the free energy profiles and the current–voltage curves obtained from the generated trajectories reproduce with good accuracy results obtained in molecular dynamics simulations.

The charged particles of which matter is composed move when an external electric field is applied, and their changed distribution is traditionally described in terms of a polarisation field. For insulators, it is usually possible to define a relative permittivity (dielectric constant) to quantify the material’s responsiveness to the electric field. In ion channels, for example, the protein walls and the water are usually treated as dielectric continua with relative permittivities of around 2 and 80, respectively. This approach can be very helpful and revealing, but it involves greater approximation than spatial averaging because, as R.S. Eisenberg points out [5], the material’s response to the electric field may be both nonlinear and time-dependent. In order to accommodate such phenomena, while simultaneously challenging physicists to review their knowledge of electromagnetism in biological dielectrics, he proposes and discusses an apparently minor change in Maxwell’s first equation. It produces a major consequence when joined with Maxwell’s second equation in that conservation of total current (including the displacement current) then emerges as a general principle. In one-dimensional systems like ion channels or electronic circuit components, the consequences are profound: there, total currents are equal at all locations at any given time, so the space variable does not appear in the description of total current.

There follow two papers reporting MD simulations of ion currents in biological and artificial channels. First, S.M. Cosseddu et al. [6] present an extended MD-based analysis of ion motion within the KcsA channel. They reveal complicated patterns of potassium currents that are governed by the structural variability of the selectivity filter. They show that ion motion involves the complex dynamics of a strongly correlated network of residues and water molecules. Intriguing features of self-organisation and readjustment of the network are analysed statistically and discussed in detail.

Secondly, we note that ionic transport in nano- to subnano-scale pores is highly dependent on translocation barriers and potential wells. These features in the free-energy landscape are primarily the result of ion dehydration and electrostatic interactions. For pores in atomically thin membranes, the ionic dynamics both inside and outside the geometrical volume of the pore can be critical in determining its transport properties. S. Sahu and M. Zwolak [7] examine regimes of transport that are highly sensitive to pore size due to the interplay of dehydration and interaction with pore charge, where picometer changes in the size (e.g., due to a minute strain), can lead to a large change in conductance.

We have already remarked upon the crucial importance of water, the electric double-layer, water-layering, polarisation, and the resultant changes of local dielectric permittivity at the entrances of nanopores. Another approach to this problem is illustrated in the paper by T.-L. Horng [8]. Starting from the classical Helmholtz free energy functional for an electrolyte, including the solvation energies for anions and cations, the author follows the Bikerman modification by adding an entropy term to the functional, and he then extends the Bikerman approach by introducing ion-size-specific corrections to the theory.

The approach based on density functional theory (DFT), which works well near charged walls and in bulk electrolytes, can be extended to the analysis of the orientational ordering of water dipoles in membrane nanotubes. M. Drab et al. [9] analyse water ordering in nanotubes by minimising the corresponding Helmholtz free energy functional, also including the orientational entropy contribution of water dipoles, and deriving the modified Langevin–Poisson–Boltzmann (MLPB) model of the electric double-layer. The MLPB equation is solved in cylindrical coordinates to determine the spatial dependences of the electric potential, relative permittivity, and average orientations of water dipoles within charged tubes of different radii. Results show that for tubes of large radius, the macroscopic (net) volume charge density of co-ions and counterions is zero on the geometrical axis. This is attributed to effective electrolyte charge screening in the vicinity of the charged inner surface of the tube. For tubes of small radius, the screening region extends into the whole inner space of the tube, leading to non-zero net volume charge density and non-zero orientational ordering of water dipoles near the axis.

The DFT results mentioned above are examples of statistical physics yielding insight into the function of ion channels and nanopores. This theme is continued and extended, first by Gibby et al. [10], who apply their recent derivation of an effective grand canonical ensemble and linear response theory of ion channels to analyse the conduction of the bacterial NaChBac selectivity filter. The authors compare their theory to experimental current–voltage and current–concentration dependences for a single channel and for a whole cell. They find that the statistical theory in the linear response regime correctly predicts many important properties of the NaChBac filter, including the concentration dependence of the reversal potential and the current–voltage relations. They also show that the theoretical results are consistent with MD simulations of the filter population at each binding site.

Secondly, the analysis of quantum mechanical effects in ion channels is another important direction, supported by the extended capabilities of modern quantum mechanics/molecular mechanics simulations. In this respect, interesting perspectives are opened by mapping the statistical mechanics of ion channels onto an effective quantum mechanics. Such investigations are reviewed by T. Gulden and A. Kamenev [11], who study the dynamics and thermodynamics of ion channels, considered as effective 1D Coulomb systems whose statistical mechanics is dominated by entropic effects that may be taken accurately into account by mapping onto an effective quantum mechanics. The corresponding semiclassical calculations for non-Hermitian Hamiltonians are conducted by applying tools from algebraic topology. The relationship of the solutions to the thermodynamics and correlation functions of multivalent solutions within long water-filled channels is discussed.

The actual properties of real nanopores are, of course, discovered by experiment, which has been leading the research in this area, especially since the discovery of the structure of the KcsA channel. In our Special Issue, experimental insight is provided by two of the leading research groups in the field.

O. Fedorenko et al. [12] discuss the properties of voltage-gated sodium channels (Navs). These channels play fundamental roles in eukaryotes but lack structural resolution, which renders understanding their structure–function relationships a challenging problem. Bacterial Navs, representing simplified homologues of their eukaryotic counterparts, have enabled both structural resolution and electrophysiological characterisation. However, their homotetrameric structure leads to an EEEE locus in the SF that is at odds with the DEKA locus of eukaryotic Navs. Indeed, prokaryotic Navs have long been considered more similar to eukaryotic calcium channels (Cavs) than to Navs, leading to the formulation of the “EEEE paradox”. This was arguably solved by Kaufman et al. by the realisation that there is a critical D residue close to the EEEE ring of eukaryotic Cavs generating an effective EEEED locus of charge −5e. Fedorenko et al. present a follow-up of a previous study, aimed at mimicking the SF of eukaryotic Navs by engineering radial asymmetry into the SFs of bacterial channels. This goal was pursued with two approaches: co-expression of different monomers of the NaChBac bacterial channel in mammalian cells to induce the random assembly of heterotetramers, and the concatenation of four bacterial monomers to form a concatemer that can be targeted by mutagenesis on specific strands of the SF, thereby introducing asymmetry. Patch-clamp measurements and MD simulations showed that an additional gating charge in the SF leads to a significant increase of Na${}^{+}$ and a modest increase in Ca${}^{2+}$ conductance in the NavMs concatemer, in agreement with the behaviour of the population of random heterotetramers with the highest proportion of channels with charge −5e. This study confirms that, although the charge at the SF is important, it is not the only factor affecting conduction and selectivity. It also offers new tools extending the use of bacterial channels as models of eukaryotic ones.

The work by A. Chernev et al. [13] reviews the most promising approaches to the fabrication of artificial nanofluidic devices capable of reproducing the properties of single ion channels. It is shown that modern technologies have great potential in allowing one to test various theoretical models of ion channels. The review aims to highlight ionic Coulomb blockade—the phenomenon which (see above) can often be a key player in ion channel selectivity. The authors discuss the most critical obstacles associated with these studies, and suggest possible solutions to further advance the field.

The rapid interdisciplinary advances in nanotechnology can be characterised as the beginning of a new industrial revolution, where novel devices and materials are fabricated and controlled on the atomic level. Ion- and water-selective nanopores represent an important frontier in these advances.

The selected papers in this Special Issue provide both a snapshot of the present as well as strong indications of how the subject is likely to evolve over the coming years. We may, for example, anticipate major developments in the theory at a fundamental level, based on statistical mechanics and quantum mechanics; substantial improvements in “intermediate-level” theories like PNP, modified CKE, and DFT which promise quantitative predictions of the properties of real channels; as well as much faster and more capacious MD modelling of larger ensembles of atoms on longer timescales, more accurate due to use of polarisable force fields and QM/MM, encompassing gating and permeation events at a statistically useful level. This progress is expected to lead to the first-principles design and fabrication of structures optimised for many important applications including ion pumps, energy harvesting, and field-effect ionic transistors as well as those mentioned at the beginning. Many of these will require theory and experiment on small scales where disciplinary distinctions have mostly faded away, but where physics predominates.

An additional impulse propelling these developments forward is expected due to the fusion of physics-based approaches with artificial intelligence. The latter has already been proven to be very useful for the accelerated learning of the force fields in MD, as well as for the reconstruction of the potentials of the mean force and neural-network-based discovery of partial differential equations. Remarkably, it also underlies a recent breakthrough in the solution of the protein-folding problem.

## Data Availability

Not applicable.
